# *Haemophilus influenzae*-induced epididymitis: a rare case report

**DOI:** 10.1097/MS9.0000000000002834

**Published:** 2025-01-21

**Authors:** Dev Patel, Luke Walker, Nalini Patel, Tirath Patel, Rajesh Perumbilavil Kaithamanakallam

**Affiliations:** a Medical School −Lokmanya Tilak Municipal Medical College, Mumbai, Maharashtra, India; b Hospitalist, Houston Healthcare, Warner Robbins, Georgia; c Infectious Medicine Consultant, Houston Healthcare, Warner Robbins, Georgia; d Trinity Medical Sciences University School of Medicine, St Vincent, Saint Vincent and the Grenadines; e American University of Antigua, Antigua and Barbuda

**Keywords:** atypical epididymitis, atypical hemophilus influenzae infection, hemophilus influenzae induced epididymitis, scrotal abscess

## Abstract

**Introduction and importance:**

Epididymitis is a common urological condition typically caused by *Chlamydia trachomatis* or *Neisseria gonorrhoeae*, with rare complications like abscess formation. *Haemophilus influenzae*, usually linked to respiratory infections, is an uncommon cause. This report discusses a 33-year-old male with *H. influenzae* epididymitis, bacteremia, and a positive NAAT for *Chlamydia*, presenting a rare and complex clinical scenario. His history of vasectomy adds further intricacy to the case. This highlights the importance of recognizing atypical pathogens, conducting thorough diagnostic evaluations, and tailoring management strategies for unusual presentations of epididymitis.

**Case presentation:**

A 33-year-old male with GERD and a history of vasectomy presented with 1 day of fevers, chills, and worsening left-sided scrotal pain. Examination showed localized scrotal tenderness and a temperature of 100.9°F. Blood cultures revealed *H. influenzae*, and NAAT confirmed *Chlamydia* with negative *Gonorrhea* testing. Ultrasound suggested epididymitis with a possible abscess, while CT showed scrotal edema without a drainable collection. Follow-up imaging maintained concerns for an abscess. Treated with Rocephin and Doxycycline, the patient showed clinical improvement, with plans for serial imaging to monitor resolution and ensure full recovery.

**Clinical discussion:**

This case highlights the challenges of managing epididymitis caused by the atypical pathogen *H. influenzae*. Rare in genitourinary infections, its detection raises questions about pathogenesis, possibly linked to the patient’s vasectomy altering local immunity. Co-infection with *Chlamydia* may have exacerbated localized inflammation. Serial imaging, particularly ultrasound, proved critical in identifying an abscess overlooked by CT. Management involved targeted antibiotics, careful monitoring of blood cultures, and follow-up imaging to prevent complications. The case underscores the importance of a broad differential diagnosis and tailored treatment in managing rare presentations of epididymitis.

**Conclusion:**

This report highlights a rare case of *H. influenzae*-induced epididymitis with bacteremia and co-existing *Chlamydia* infection in a post-vasectomy patient. It underscores the importance of recognizing uncommon pathogens, especially in patients with unique risk factors like vasectomy. Serial imaging was crucial for identifying complications such as abscess formation. Early diagnosis and targeted therapy were essential in preventing serious outcomes like chronic inflammation or abscess rupture. This case contributes to the limited literature on atypical epididymitis, emphasizing the need for a multidisciplinary approach in managing complex infections effectively.

## Introduction

Epididymitis is an inflammation of the epididymis, which is a common urological condition that typically affects sexually active men under 35 and older men with urinary tract pathology. It is often caused by sexually transmitted infections (STIs) like *Chlamydia trachomatis* and *Neisseria gonorrhoeae* or by enteric pathogens in older populations. Epididymal abscesses, though uncommon, represent a severe complication of epididymitis and pose significant diagnostic and therapeutic challenges^[1]^.

This case report describes a 33-year-old male with a complex presentation of epididymitis complicated by an abscess, bacteremia with *Haemophilus influenzae*, and positive *Chlamydia* NAAT. The patient’s history, including a vasectomy, adds further complexity to the clinical scenario. This report aims to highlight the diagnostic process, therapeutic interventions, and clinical decision-making involved in managing such an intricate case. This report underscores the importance of considering atypical pathogens in epididymitis and the need for thorough diagnostic imaging and follow-up.

## Case presentation

**Figure 1.**
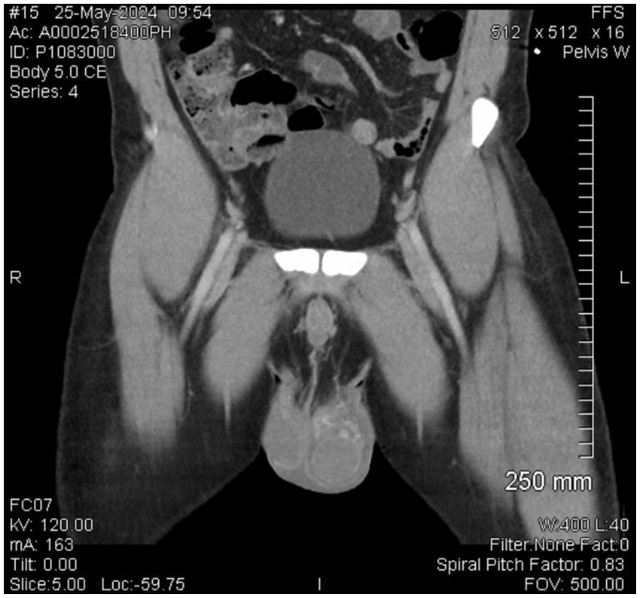
CT scan of the pelvis showing scrotal edema involving both scrotal sacs.

**Figure 2.**
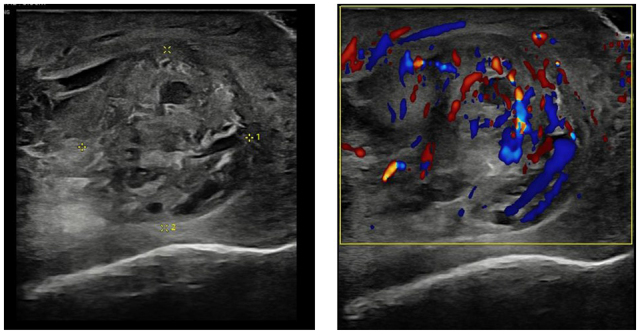
Ultrasound of the left testis showing epididymitis with a small complex fluid collection along the distal epididymis raising suspicion for an abscess.

A 33-year-old male presented to the hospital with complaints of sweats, chills, fevers, and progressively worsening left-sided scrotal pain of 1-day duration. His medical history included GERD and a vasectomy performed ten years prior. The patient reported initial symptoms of body aches, sweats, and chills, followed by the onset of scrotal pain. On examination, his vital signs showed a maximum temperature of 100.9°F. Blood cultures were positive for *H. influenzae*, and NAAT (first-morning void urine sample) for *Chlamydia* was positive, while tests for Gonorrhea were negative. Laboratory findings included elevated WBC (20 100) with neutrophilia (81.3%) and one-time elevated glucose of 125 mg/dL (results are given in the tables below). Initial scrotal ultrasound findings suggested left epididymitis with a small complex fluid collection, raising suspicion for an abscess, along with a small left-sided hydrocele and suspected left varicocele (as shown in Fig. 2). A CT pelvis confirmed scrotal edema but did not identify a drainable fluid collection (as shown in Fig. 1). A follow-up ultrasound indicated that an early phlegmon or abscess could not be entirely excluded, suggesting the need for I&D.

The patient was treated with Rocephin and Doxycycline. His blood cultures, initially positive for *H. influenzae*, were negative on subsequent testing. The treatment plan included obtaining additional blood cultures to monitor bacteremia and scheduling follow-up imaging to ensure resolution of the abscess.

## Laboratory findings

### CBC

**Table:** 

Parameter	Value
WBC	**20.1 (High)**
RBC	5.23
Hgb	14.2
Hct	44.4
MCV	84.9
MCH	27.2
MCHC	**32.0 (Low)**
RDW	12.7
Platelet count	234
MPV	10.1
Neutrophils % (auto)	**81.3 (High)**
Lymphocytes % (auto)	**8.8 (Low)**
Monocytes % (auto)	9.0
Eosinophils % (auto)	0.0
Basophils % (auto)	0.2
Neutrophils (auto)	**16.4 (High)**
Lymphocytes (auto)	1.8
Monocyctes (auto)	**1.8 (High)**
Eosinophils (auto)	0.0
Basophils (auto)	0.0
Absolute nucleated RBC	0.00
Immature granulocytes %	0.7
Nucleated RBC %	0.0
Immature granulocytes %	0.14

### CMP

**Table:** 

Parameter	Value
Sodium	140
Potassium	3.6
Chloride	101
Carbon dioxide	29
anion gap	10
BUN	12
Creatinine	1.20
Estimated creatinine clearance	107.77
GFR calculation	70
BUN/creatinine ratio	**9.6 (Low)**
Glucose	**125 (High)**
Calculated osmolality	279.4
Calcium	9.0
Magnesium	**1.6 (Low)**
Total bilirubin	0.8
AST	**8 (Low)**
ALT	29
Alkaline phosphatase	52
Total protein	6.6
Albumin	3.6
Globulin	3.0
Albumin/globulin ratio	1.2

### Microbiology

**Table:** 

Test	Result
Blood culture (day 1)	***Haemophilus influenzae* isolated from 2 samples**
Blood culture (day 2)	No growth
NAAT (first morning void urine sample)	**Chlamydia: Positive (1:64),** Gonorrhea: Negative,

### Imaging

**Table:** 

Imaging	Findings
Scrotal Ultrasound	Findings suggestive of left epididymitis with a small complex fluid collection along the distal epididymis raising suspicion for an abscess. Associated small left-sided hydrocele is noted. Left varicocele suspected.
CT Pelvis	Scrotal edema involving both scrotal sacs, left greater than right. No definite drainable fluid collection identified. Questional epididymal abscess on prior ultrasound not seen on CT.
Repeat Scrotal Ultrasound	Suspect left-sided epididymitis; an early phlegmon or abscess cannot be entirely excluded. Follow-up in 3 weeks recommended to confirm resolution.

## Discussion

Epididymitis is a frequent cause of scrotal pain in adults and typically presents with localized pain, swelling and tenderness in the epididymis, sometimes radiating to the lower abdomen and adjacent testis. Complicated cases may show swelling, fever, and tachycardia with scrotal wall erythema. Diagnosis involves history, physical examination, urinalysis, urine culture, and NAAT for *N. gonorrhoeae* and *C. trachomatis*. Ultrasonography often reveals an enlarged, thickened epididymis with increased Doppler wave pulsation.^[2]^.

However, this case presents a unique scenario with *H. influenzae* as the causative pathogen, which is unusual in the context of epididymitis. The positive *Chlamydia* NAAT further complicates the etiology suggesting a possible co-infection. The patient’s history of vasectomy is a notable aspect that may have predisposed him to this infection. While vasectomies are generally low-risk procedures, they can create subtle anatomical changes in the epididymal environment, such as increased back pressure and micro-structural alterations, potentially enhancing susceptibility to infections. These changes may disrupt the local immune environment, making it easier for pathogens like *H. influenzae* to colonize and cause inflammation. Localized immunosuppression by *Chlamydia* co-infection or anatomic abnormalities in the urinary tract could be the significant reasons for infection by *H. influenzae* ^[3]^.

The diagnostic imaging findings were critical in managing this case. The initial ultrasound suggested an epididymal abscess, which was concerning for a severe, localized infection. The subsequent CT pelvis scan did not identify a drainable fluid collection, which initially appeared reassuring. However, the follow-up ultrasound continued to raise suspicion for an abscess or early phlegmon, underscoring the importance of serial imaging in the management of epididymal abscesses. The absence of a drainable fluid collection on CT highlights the limitations of this imaging modality in detecting small or early abscesses compared to ultrasound.

The clinical implications of treating *H. influenzae* infections in the genitourinary system, particularly in epididymitis, are substantial given the rarity of this pathogen in such contexts. *H. influenzae* is primarily associated with respiratory infections, and its management in the genitourinary tract presents unique challenges due to limited clinical precedents. Clinicians must consider that atypical infections may not respond predictably to standard antibiotic regimens typically used for more common uropathogens.

This case is being reported due to the rare presentation of *H. influenzae* epididymitis and the complexity added by the patient’s positive *Chlamydia* NAAT and history of vasectomy. The management of this case required a multi-faceted approach, including targeted antibiotic therapy, close monitoring of blood cultures, and careful interpretation of serial imaging studies. This report aims to contribute to the limited literature on atypical pathogens in epididymitis and to emphasise the importance of considering a broad differential diagnosis in similar clinical scenarios.

## Literature review

*Haemophilus influenzae is* a Gram-negative coccobacillus, is more commonly known as a pathogen responsible for respiratory infections, particularly in pediatric populations, including otitis media, sinusitis, and pneumonia. The literature reveals that *H. influenzae*-induced epididymitis is an uncommon manifestation and is typically reported in adult males with underlying health conditions, particularly those with immunosuppression or anatomic abnormalities in the urinary tract. Case reports highlight that the infection may present with similar clinical features as epididymitis caused by more common pathogens including unilateral scrotal pain, swelling, and fever^[4]^. However, the rarity of the condition often leads to delayed diagnosis and treatment, as it is not initially considered in differential diagnoses.

The pathogenic mechanism is believed to involve hematogenous spread, although direct extension from adjacent sites cannot be ruled out. Diagnosis is confirmed by urine culture or epididymal aspirate culture which identifies *H. influenzae* as the causative agent. Treatment typically involves the use of antibiotics, with broad-spectrum coverage initially, followed by more targeted therapy based on culture sensitivities. The prognosis is generally favorable with appropriate antimicrobial treatment, although delayed diagnosis can result in complications such as abscess formation or chronic epididymitis^[5]^. Common pathogens include sexually transmitted organisms like *Chlamydia* trachomatis and *N. gonorrhoeae* and non-sexually transmitted organisms like Escherichia coli and other rare organisms can also cause epididymitis.

In one case study, *H. influenzae* was isolated in a 51-year-old immunocompetent male with epididymo-orchitis and concurrent bacteremia, highlighting a severe but unusual presentation. The patient’s symptoms included unilateral scrotal pain and fever, and he responded well to a course of broad-spectrum antibiotics following pathogen identification via blood cultures^[1]^. Additional reports underscore the role of *H. influenzae* in epididymitis when compounded by factors like localized immunosuppression or structural changes in the urinary tract. This association between vasectomy and a predisposition to atypical infections provides valuable insight into the clinical management of post-vasectomy complications and reinforces the need for thorough microbial investigation in such presentations.

Other atypical pathogens have been implicated in epididymitis under similarly rare conditions. For instance, Mycobacterium tuberculosis has been documented in genitourinary tuberculosis cases, particularly in immunocompromised individuals or regions where tuberculosis is endemic. A case of tuberculous epididymitis was seen in a 32-year-old male with a small lump in his left scrotum. A diagnosis of genitourinary tuberculosis was established with radiometric investigations and the isolation of the bacteria from the cartridge-based nucleic acid amplification test. He was managed conservatively with anti-tuberculous drugs for 6 months.^[6]^. Brucella species also present as a causative agent in patients exposed to infected animals or endemic areas. A 2021 study reported a case series of *Brucella melitensis* with prostatic abscess, illustrating the zoonotic potential of certain pathogens in epididymal infections. Such cases reinforce the importance of considering atypical bacterial agents when common causes are ruled out^[7]^.

These findings underscore the importance of broadening the differential diagnosis in epididymitis, particularly when symptoms persist despite standard treatment or in patients with uncommon risk factors like vasectomy. The rarity of pathogens such as *H. influenzae, Mycobacterium tuberculosis*, and *Brucella* in epididymitis requires clinicians to adopt a multidisciplinary approach and prioritize culture-specific antibiotic therapy to avoid complications such as abscess formation and chronic inflammation^[8]^.

## Conclusion

This case of *H. influenzae* -induced epididymitis, complicated by bacteremia and a positive *Chlamydia* NAAT, underscores the diagnostic and therapeutic complexities of managing atypical infections in the genitourinary system. The patient’s history of vasectomy, along with the unexpected pathogen, highlights the importance of considering procedural impact on local anatomy and immunity as potential risk factors for rare infections. This case emphasizes the need for clinicians to expand their differential diagnoses to include uncommon pathogens when typical treatment pathways prove ineffective. By considering these rare pathogens early in atypical presentations, clinicians can improve patient outcomes through timely diagnosis and targeted therapeutic interventions, reducing the risk of complications such as abscess formation and chronic inflammation.

## Data Availability

Data is publicly available.
